# Therapeutic progress in the management of triplane fractures in adolescents

**DOI:** 10.3389/fped.2026.1649784

**Published:** 2026-04-14

**Authors:** Qiqi Wei, Wenlong Ma, Guangshui Lv, Fanglong Zheng, Cheng Jing, Yanbo Guo

**Affiliations:** 1 The Department of Traumatic Orthopedics, Shandong Wendeng Orthopedic Hospital, Weihai City, Shandong Province, China; 2 The Department of Traumatic Orthopedics, Shandong Second Provincial General Hospital, Jinan City, Shandong Province, China; 3 The Department of Pediatric Orthopedics, Affiliated Hospital of Shandong University of Traditional Chinese Medicine, Jinan City, Shandong Province, China; 4 The Department of Orthopedics, Affiliated Hospital of Shandong University of Traditional Chinese Medicine, Jinan City, Shandong Province, China; 5 The First Clinical Medical School, Shandong University of Traditional Chinese Medicine, Jinan City, Shandong Province, China; 6 The Department of Joint Orthopedics, Affiliated Hospital of Shandong University of Traditional Chinese Medicine, Jinan City, Shandong Province, China

**Keywords:** adolescent, dignosis, transitional fracture, treatment, triplane fracture

## Abstract

The triplane fracture is a complex, idiosyncratic, and relatively rare type of specialized fracture of adolescents. The fracture type of triplane fractures is determined by the mechanism of injury and the degree of closure of the epiphyseal plate. The absence of a universally accepted framework for categorizing triplane fractures increase the likelihood of being underdiagnosed. By utilizing x-rays and CT scans, it is possible to gain a comprehensive and precise understanding of fracture characteristics, which can then be used to identify other concurrent injuries and provide precise guidance for treatment. There is still no broad consensus on its treatment scheme. Without proper treatment, it may cause adverse consequences such as osteoarthritis and angular deformity. This article summarizes the clinical research conducted on the diagnosis and treatment of triplane fractures, to enhance clinicians' comprehension of this unique fracture pattern, augment diagnostic precision, and enhance treatment results.

## Introduction

Triplane fractures are distinctive fractures characterized by fracture lines that are visible across the sagittal, coronal, and horizontal (axial) planes, extending through the metaphysis, epiphyseal plate (growth plate), and the articular surfaces of the epiphysis. This complex fracture pattern was initially described by Bartl in 1957, with the term “triplane fracture” later being coined by Lynn in 1972 (1–3). As the skeleton transitions from childhood to adulthood, it undergoes maturation and gradual closure of growth plates. Titze was the first to characterize a specific type of ankle fracture occurring during this critical period as a “transitional fracture” (4). Triplane fractures, which manifest during this transitional stage of skeletal development, are thus also categorized under the umbrella of transitional fractures (5). As a distinct fracture pattern, triplane fractures cannot be adequately classified using the Salter-Harris typing system alone, and they typically result from medium- to low-energy traumatic incidents (6, 7). The most prevalent causes of triplane fractures are falls.

**Figure 1:**
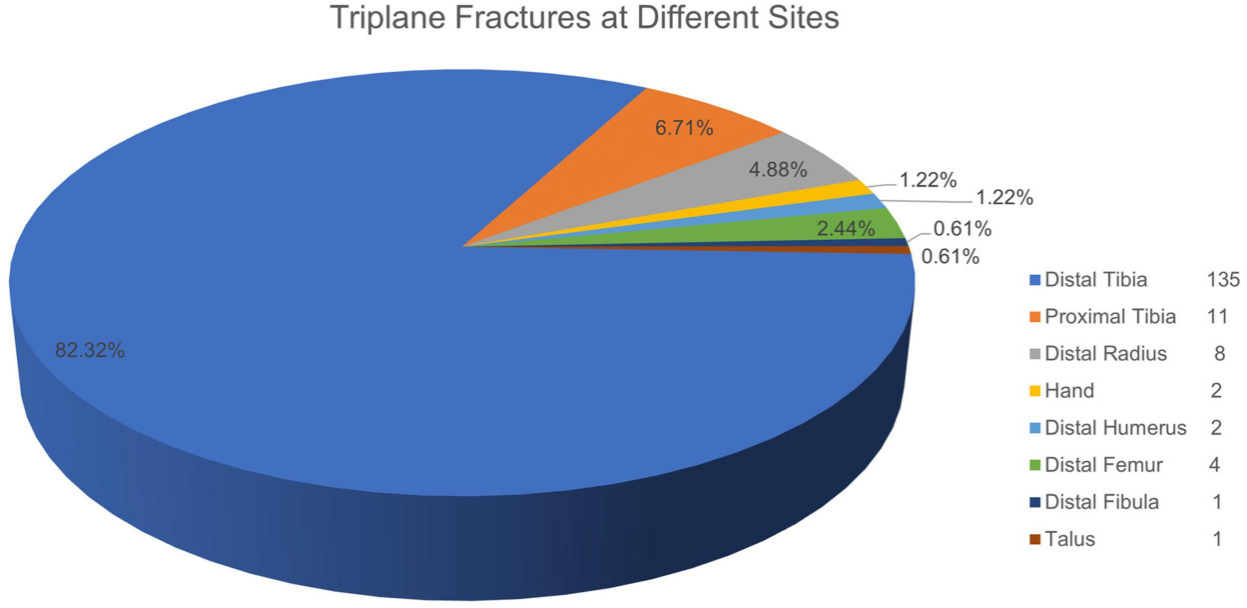
Distribution of literature in the pubMed database on triplane fractures by anatomical location. We conducted a search in the PubMed database using the search terms “triplane fracture”, “transitional fracture” and “adolescent”. The search time span covers from the establishment of the database up to June 2025. The highest proportion of literature focuses on triplane fractures of the distal tibia, followed by those of the proximal tibia and distal radius. Triplane fractures in other anatomical locations account for the smallest percentage, with only a few cases documented in the literature.

Triplane fractures typically occur during the transitional phase between puberty and skeletal maturity, most commonly affecting adolescents aged 12–15 years, with a mean age of 13.4 years. These fractures occur more frequently in males, who also exhibit a significantly higher average age at injury (14.2 ± 0.6 years) compared to females (12.6 ± 0.9 years) (8–13). The distal tibia is the most commonly affected site for triplane fractures, with limited documented evidence of their occurrence in other anatomical regions. However, rare and sporadic cases have been reported in the proximal tibia, distal femur, distal radius, distal humerus, hand (the proximal phalanx), talus, and distal fibula (14–23). To date, triplane fractures of the distal tibia constitute approximately 5%–10% of all ankle fractures and 15% of epiphyseal injuries among adolescents (8, 24, 25). Fractures involving the proximal tibial epiphyseal cartilage account for merely 1%–3% of all epiphyseal injuries (15, 20, 26). However, to date, no large-scale epidemiologic surveys have comprehensively examined triplane fractures across all anatomical sites in adolescents. Furthermore, a review of the PubMed database reveals that 82.32% of the literature focuses on triplane fractures of the distal tibia, while 6.71% and 4.88% pertain to those of the proximal tibia and distal radius, respectively (Figure 1). Triplane fractures in other body regions are documented almost exclusively in case reports and remain exceedingly rare.

This paper summarizes clinical research on the diagnosis and management of triplane fractures to enhance clinicians' comprehension of this distinct fracture pattern, refine diagnostic accuracy, and optimize treatment outcomes.

## Pathogenesis

During adolescent growth and development, the epiphyseal plate remains open, and the triplane fracture pattern occurs because the physis is in the process of closing (27). Triplane fractures represent a particularly complex and unique injury pattern, with the mechanisms of damage in various anatomical regions not yet fully elucidated. Nevertheless, the physiological and anatomical factors contributing to triplane fractures are well-recognized. As the bone matures normally, the epiphyseal plate or growth plate progressively thins, followed by partial and eventual complete closure. This process leads to the disappearance of the epiphyseal plate and cessation of endochondral ossification (28). Theoretically, triplane fractures can occur at any partially ossified epiphysis. However, the spatial pattern of epiphyseal plate closure varies across different anatomical regions and is not fully elucidated at skeletal maturity, complicating efforts to study the mechanisms of triplane fracture damage.

**Figure 2:**
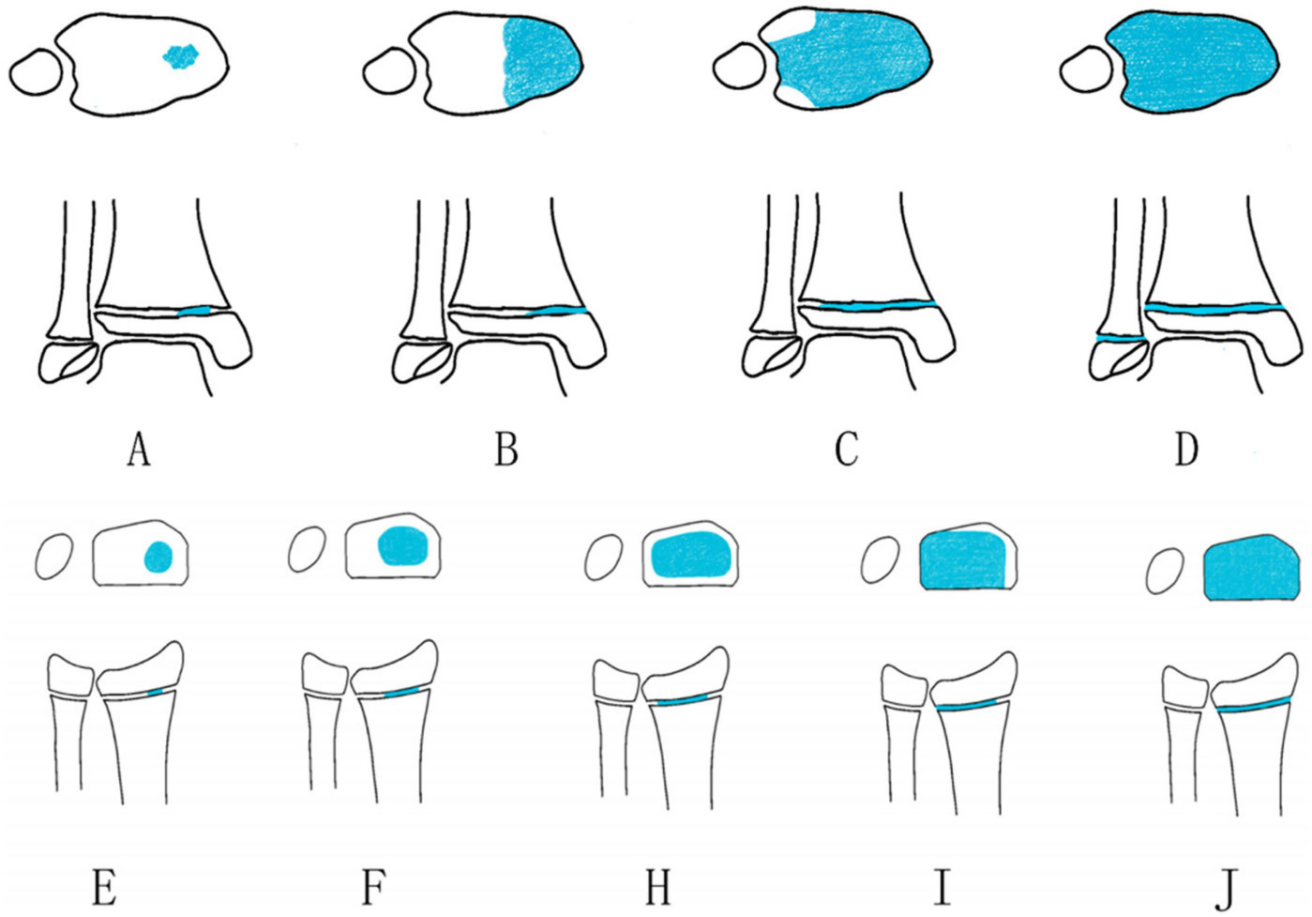
Schematic representation of distal tibial epiphyseal plate closure: ossification initiates at Kump's augmentation **(A)** and proceeds in a counterclockwise direction, first reaching the anteromedial region **(B)**, then gradually advancing toward the posteromedial region **(C)**, and finally concluding with closure at the lateral margin of the epiphyseal plate **(D)** schematic representation of distal radial epiphyseal plate closure: ossification begins centrally within the radius **(E)** and progresses medially and laterally **(F,H)**, with medial ossification occurring at a higher rate than lateral ossification. Closure occurs first at the medial margin **(I)** and is completed at the lateral margin **(J)**

For the most common clinical presentation—triplane fractures of the distal tibia—the closure of the distal tibial epiphyseal plate is a prolonged process, spanning up to 18 months (typically occurring between ages 12 and 15). During this period, the epiphyseal plate undergoes asymmetric closure, with ossification initiating centrally (a process referred to as Kump's augmentation) and progressing in an anticlockwise direction from the anteromedial to posteromedial regions, culminating in the closure of the lateral margin (Figures 2A–D). This sequential spatial pattern of epiphyseal plate maturation in the distal tibia heightens the risk of transitional injuries in adolescent patients, potentially leading to Tillaux fractures or triplane fractures. The stage of epiphyseal plate closure at the time of injury influences the fracture type, as variations in ossification patterns result in distinct fracture morphologies. As the growth plate matures and calcifies, the bone assumes a more “adult-like” configuration, and the fracture line shifts laterally as the calcified zone of the growth plate expands. Thus, the more advanced the growth plate maturation, the more lateral the fracture tends to be. Triplane fractures have also been reported in the proximal tibia and distal femur, where physiological closure of the epiphyseal plate similarly initiates centrally and propagates toward the periphery (29). Margalit et al. (30) conducted a retrospective study involving magnetic resonance imaging (MRI) imaging of the knee in 165 adolescent patients (aged 6–19 years, mean 14.7 years) to evaluate the correlation between physeal maturation stage and age across eight anatomical regions, including the proximal tibia, proximal fibula, and distal femur. The study revealed that the spatial sequence of epiphyseal maturation in these eight knee regions was consistent between males and females. Although closure of the epiphyseal plate occurred at an earlier age in females, it was characterized by homogeneity, in contrast to the asymmetric closure observed in the distal tibial epiphyseal plate. The lower incidence of triplane fractures in the proximal tibia compared to the distal tibia is attributed to the more symmetrical closure of the proximal tibial epiphyseal plate and the influence of surrounding ligaments, which redirect stress to the metaphysis rather than the epiphyseal plate at this site (8, 15, 16, 26). Ossification of the distal radial epiphyseal plate typically initiates uniformly from the center and progresses toward the medial and lateral sides, with the medial side usually ossifying first (Figures 2E–J). This entire process is completed within less than a year. A 2011 study involving serial MRI assessments of the distal radial epiphyseal plate in 22 adolescents demonstrated that ossification of the distal radial growth plate begins centrally and concludes at the dorsal margin of the radius (31). The homogeneity of both the closure sequence and the closure cycle of the epiphyses may account for the relatively lower frequency of transitional injuries in this region compared to the ankle.

However, Prijs (32) et al.'s study investigated the pathoanatomy of distal tibial triplane fractures, challenging the classic presentation that physeal closure dictates the overall fracture pattern. A fracture mapping analysis demonstrated that, regardless of age and sex, fracture lines consistently converged in a characteristic Y-pattern, which remained unchanged with physeal closure. Furthermore, the study found that fracture lines typically occurred between the anterior and posterior inferior tibiofibular ligaments and the medially fused physis or deltoid ligament, underscoring the pivotal role of ligament attachments in shaping the fracture patterns of these injuries.

The complexity of adolescent triplane fractures arises from the interplay between the growth plate's closure pattern and the traumatic forces incurred during injury. As the epiphyseal plate is only partially ossified and closed, the traumatic energy is transmitted from the ossified regions of the epiphyseal plate to the unossified portions, resulting in a fracture (33). External rotation of the ankle joint is the most prevalent mechanism underlying triplane fractures of the distal tibia. Under torsional forces from external rotation, a simultaneous fracture occurs at the anterolateral epiphyseal plate of the distal tibia, which remains attached to the posterior portion of the distal tibial metaphysis. The fracture line traverses the articular surface of the distal tibia in the sagittal plane, resulting in a characteristic two-part fracture configuration. Other variants of triplane fractures often evolve from this foundational pattern (34). If external rotation of the ankle is further intensified, it may result in an oblique fracture of the fibula. Notably, approximately 50% of triplane fractures of the distal tibia are accompanied by an ipsilateral fibula fracture, suggesting that a higher magnitude of traumatic energy likely leads to more extensive combined injuries (13). Classical triplane fractures and Tillaux fractures are characteristic of adolescent ankle fractures occurring in the supinated position, particularly in injuries involving supination-external rotation mechanisms (35, 36). At the same time, according to the Lauge-Hansen classification, paediatric triplane ankle fractures and adult trimalleolar fractures exhibit common features (37).

Additional mechanisms have also been documented (8, 11, 13, 38). Triplane fractures of the proximal tibia are predominantly caused by high-energy injuries, such as motorcycle or bicycle accidents, or falls during activities like skiing or soccer. These fractures may be accompanied by a fracture of the tibial tuberosity epiphyseal plate, often resulting from a sudden quadriceps muscle contraction or during forceful passive knee flexion, potentially combined with rotational forces (4, 39). In contrast, injuries to the distal radius are usually caused by axial compression rather than torsional violence. There are relatively few reports of triplane fractures in other parts of the body, and their causative factors and mechanisms of injury need to be further investigated (Table 1). Interpretation of the temporal and spatial sequence of closure of the epiphyseal plate and the cycle of closure is essential to study the injury mechanism of transitional fractures.

**Table 1:** Review on triplane fractures in rare sites. Satisfactory outcome was defined as the return to normal activities and absence of functional limitations.

Triplane Fractures in Rare Sites									
Site	Author	Year	Gender	Age	Mechanism of injury	Treatment	Follow-up (months）	Complications	Outcome
Proximal tibia	Conroy (40)	2000	F	14	Fall	CRIF	Long-term	None	Satisfactory
	Hermus (41)	2003	M	17	Soccer ball	ARIF	6	None	Satisfactory
	Neilly (42)	2015	M	14	Soccer ball	ORIF	3	None	Satisfactory
	Strelzow (26)	2017	F	13	Snowboarding	ARIF	1.5	None	Satisfactory
	Aymen (15)	2019	M	12	Fall from height	CRIF	12	None	Satisfactory
	Lehreitani (20)	2019	M	16	Bicycle	CRIF	12	None	Satisfactory
	Li dasong (43)	2021	M	15	Basketball	ORIF	12	None	Satisfactory
	Whiting (16)	2021	M	13	Fall	ARIF	1.5	None	Satisfactory
Distal radius	García-Mata (44)	2006	M	13	Traffic accident	Plaster cast	36	Partial growth retardation	Satisfactory
	Pearce (45)	2011	M	14	Fall	Plaster cast	9	None	Satisfactory
	Mingo-Robinet (10)	2014	M	15	Fall	ORIF	9	Partial growth retardation	Satisfactory
	Parkar (21)	2014	M	15	Fall	Plaster cast	24	Partial growth retardation	Satisfactory
	Rauer (14)	2020	F	17	Soccer ball	Plaster cast	12	None	Satisfactory
			M	18	Motorcycle	ORIF			
			M	16	Skateboard	ORIF			
	Kurland (46)	2021	M	16	Fall	Plaster cast	36	None	Satisfactory
Hand	Garcia Mata (47)	1999	M	12	-	Plaster cast	1	None	Satisfactory
	Chin (48)	1999	M	14	Slider (baseball)	ORIF	24	None	Satisfactory
Distal humerus	Peterson (49)	1983	F	11	Fall	ORIF	5	None	Satisfactory
	Ichihara (50)	2024	M	6	Fall	ORIF	18	None	Satisfactory
Distal femur	Gosselin (51)	2005	M	9	Fall	ORIF	-	None	Satisfactory
	Masquijo (17)	2011	M	13	Traffic accident	ORIF + arthroscopy	24	Joint adhesions; Limb shortening.	Satisfactory
	Spagnolo (52)	2016	F	8	Traffic accident	ORIF	84	None	Satisfactory
	Carroll (53)	2020	F	13	Soccer ball	ORIF	12	Partial growth retardation	Satisfactory
Distal fibula	Ruffing (18)	2010	F	14	Fall	ORIF	5	None	Satisfactory
Talus	Monestier (22)	2021	F	13	Fall	ORIF	6	None	Satisfactory

“-”, Not mentioned. CRIF, closed reduction and internal fixation; ARIF, arthroscopically assisted reduction and internal fixation; ORIF: open reduction and internal fixation.

## Diagnosis

The typical clinical presentation of a triplane fracture includes pain and swelling at the fracture site, along with functional impairment. However, if the injury is in an obscure area (on the x-ray where the fracture line may be difficult to visualize clearly due to overlapping anatomical structures), the fracture displacement may be inconspicuous. More critically, most triplane fractures involve complex fracture lines: coronal fractures at the epiphyseal end, transverse fractures at the epiphyseal plate, and intra-articular fractures in the sagittal plane of the epiphyseal plate. Consequently, relying solely on frontal and lateral x-rays to diagnose and classify a triplane fracture is challenging, as these images may not accurately depict the true fracture displacement, thereby increasing the risk of misdiagnosis or underdiagnosis (54, 55). Consequently, while x-rays serve as an initial diagnostic tool, additional oblique and mortise view radiographs are recommended to better visualize fracture displacement and facilitate accurate assessment (10). In 2011, Gourineni et al. (56) proposed that widening of the medial ankle spaces could indicate fracture displacement. Specifically, 86% of transitional fractures (19 out of 22) exhibited a medial ankle gap widening of more than 1 mm (mean: 2.53 mm, range: 1–9 mm), which corresponded to the magnitude of intra-articular fracture displacement. Additionally, metaphyseal fracture displacement observed on lateral x-rays may predict intra-articular diastasis in juvenile triplane ankle fractures. The study demonstrated that metaphyseal displacement exceeding 1 mm strongly suggests the likelihood of intra-articular displacement, thereby recommending advanced imaging for further evaluation (57). Recently, the study conducted by Sang et al. (58) demonstrated that an increased articular displacement observed on both the mortise and lateral radiographic views, as well as a widened tibiofibular clear space on the mortise view, are correlated with greater displacement in transitional ankle fractures. Identifying specific radiographic parameters or reliable radiographic predictors of displacement could enable surgeons to employ computed tomography (CT) imaging more selectively in pediatric patients.

**Figure 3:**
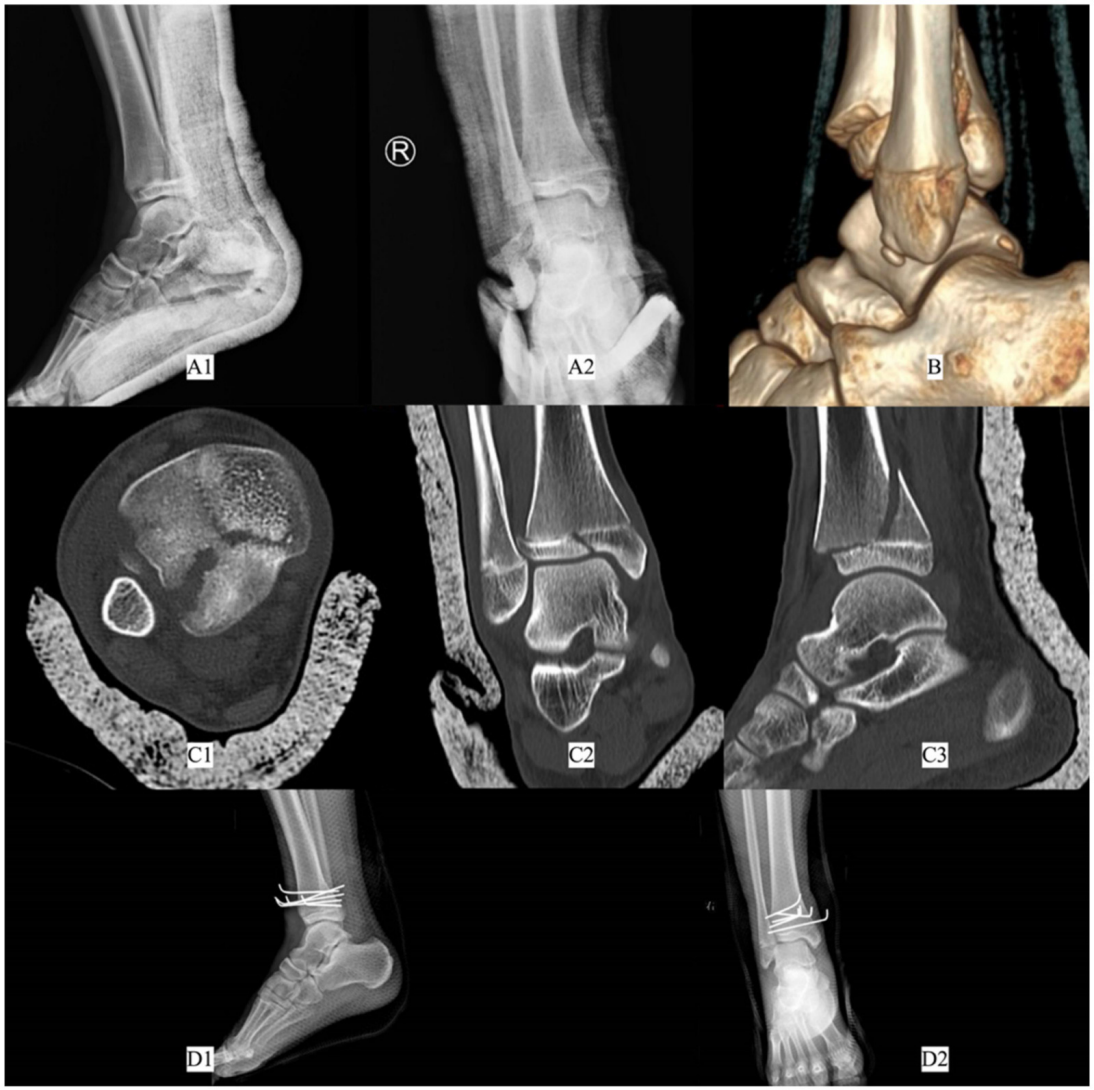
**(A)** initial x-ray after closed reduction. **(B)** Three-dimensional reconstruction CT after closed reduction. **(C)** Axial, coronal and sagittal image of initial CT after closed reduction. **(D)** Final radiography after surgery.

The routine use of CT in the evaluation of triplane fractures was once a subject of controversy, particularly given that patients with immature skeletons are subjected to higher doses of radiation as well as the higher cost containment during CT scans (59–61). However, given the high incidence of underdiagnosis with conventional x-rays, numerous scholars have advocated for the incorporation of CT scans to enhance the accuracy of triplane fracture assessment (59–61). A nationwide questionnaire from the German Trauma Society revealed that 55.62% of respondents opt for CT imaging as the cross-sectional imaging modality when suspecting fractures, while 95.05% (499/525) deem CT imaging to be beneficial for diagnosing triplane fractures (62). CT scans with 3D reconstruction provide detailed visualization of fracture characteristics, including the direction, degree of displacement, fracture line trajectory, and the number of fracture fragments, thereby facilitating a more comprehensive understanding of the injury pattern presented. This enables more accurate fracture classification and a clearer understanding of the fracture's three-dimensional spatial configuration. A representative case of x-rays and CT scans is illustrated below (Figure 3). Hadad et al. (38) employed axial CT scans to analyze distal tibial triplane fractures in 35 patients, aiming to elucidate the fracture line features in the metaphyseal and epiphyseal regions of the distal tibia on the axial plane. Their findings revealed that, in the metaphysis, fractures most commonly occurred as mediolateral lines in the posterior metaphysis, with anterior and anterolateral metaphyseal involvement being uncommon. Conversely, on the epiphyseal side, all fracture exits and lines were localized to the anterior epiphyseal plate, extending toward the posteromedial epiphyseal plate. Eismann et al. (63) conducted a study to compare the reliability of x-ray and CT in assessing the pattern and treatment of ankle triplane fractures. The findings revealed that 38% and 46% of reviewers adjusted their assessments of the number of fracture fragments and fracture pattern classification, respectively, following CT evaluation. Notably, among cases initially assessed as having ≤2 mm of displacement via x-ray, 39% were found to have >2 mm of displacement upon CT assessment. Conversely, 7% of cases initially classified as having >2 mm of displacement by x-ray were actually found to have ≤2 mm of displacement upon CT review. These discrepancies influenced treatment decisions, with 27% of cases requiring a shift to surgical intervention and 41% necessitating substantial modifications to the surgical plan, including changes in the number or direction of screw placement. These results underscore the value of CT imaging in providing precise anatomical guidance for screw placement, thereby facilitating more accurate, rational, and effective treatment planning. Adequate preoperative evaluation and planning, informed by CT, are critical for optimizing treatment outcomes and prognostic outcomes (64).

Triplane fractures may be associated with concomitant ligamentous injuries or fractures at other anatomical sites, such as the ipsilateral tibial shaft, fibula, or ulna (14, 65, 66). In such cases, these combined injuries may be overlooked, leading to underdiagnosis of triplane fractures. Specifically, when accompanied by an ipsilateral tibial shaft fracture, the more pronounced clinical manifestations and displacement of the tibial shaft fracture tend to attract attention, whereas distal tibial epiphyseal injuries with less obvious displacement or more subtle presentations are often neglected. Routine x-ray examinations alone are prone to misdiagnosis, as the overlap between the lateral x-ray view and the fibula further increases the risk of diagnostic errors. In the study conducted by Schaeffer et al. (67), 22 out of 515 pediatric tibial shaft fractures (approximately 4.3%) were found to have concomitant ipsilateral distal tibial fractures. Among these cases, transitional fractures—comprising 11 cases of triplane fractures and 2 cases of Tillaux fractures—accounted for up to 59% of the patients. Notably, 8 patients with concomitant ipsilateral tibial shaft and distal tibial fractures were underdiagnosed. The occurrence of an oblique or spiral fracture in the lower one-third of the tibial shaft was associated with a higher risk of concomitant transitional fractures and an increased likelihood of missed diagnoses (68). Based on the foregoing analysis, CT should be considered when initial x-rays are inconclusive or when a more detailed assessment of fracture morphology, particularly in complex cases, is required. It is especially useful for identifying subtle fractures, assessing the extent of articular involvement, and guiding surgical planning. When triplane fractures are suspected based on clinical presentation and following initial x-ray, CT scans with 3D reconstruction are strongly recommended to facilitate accurate diagnosis and appropriate treatment decision-making.

If warranted, MRI can be utilized to exclude potential concomitant injuries, such as ligament, meniscal, or cartilage damage near the fracture site (15, 69). In cases of distal tibial triplane fractures, tears of the deltoid ligament and injuries to the lower tibiofibular syndesmosis have been reported (70). Additionally, MRI can clearly visualize periosteal entrapment at the fracture site, which may impede reduction, thereby aiding in the rational selection of treatment options. Park et al. (71) analyzed the correlation between fracture morphology and periosteal entrapment in patients with distal tibial epiphyseal fractures using MRI, revealing that periosteal entrapment was consistently observed at the displaced anterolateral corner fracture of the distal tibia in all patients with triplane fractures.

## Fracture classification

Triplane fractures represent an intricate and distinct pattern of epiphyseal injury, characterized by a combination of different Salter-Harris types II, III, and IV fractures in both the sagittal and coronal planes. However, strictly speaking, triplane fractures cannot be adequately classified solely by the Salter-Harris classification system. Currently, there is no universally accepted clinical classification system for triplane fractures at all anatomical sites. The incidence of triplane fractures occurring outside the distal tibia is relatively low, and their characteristics vary during epiphyseal fusion and closure, further complicating classification efforts. Even for the relatively more common triplane fractures of the distal tibia, a standardized classification system remains elusive (60). Currently, triplane fractures are predominantly classified based on the number of fracture fragments, the location of fracture lines, and their impact on the joint surface.

**Figure 4:**
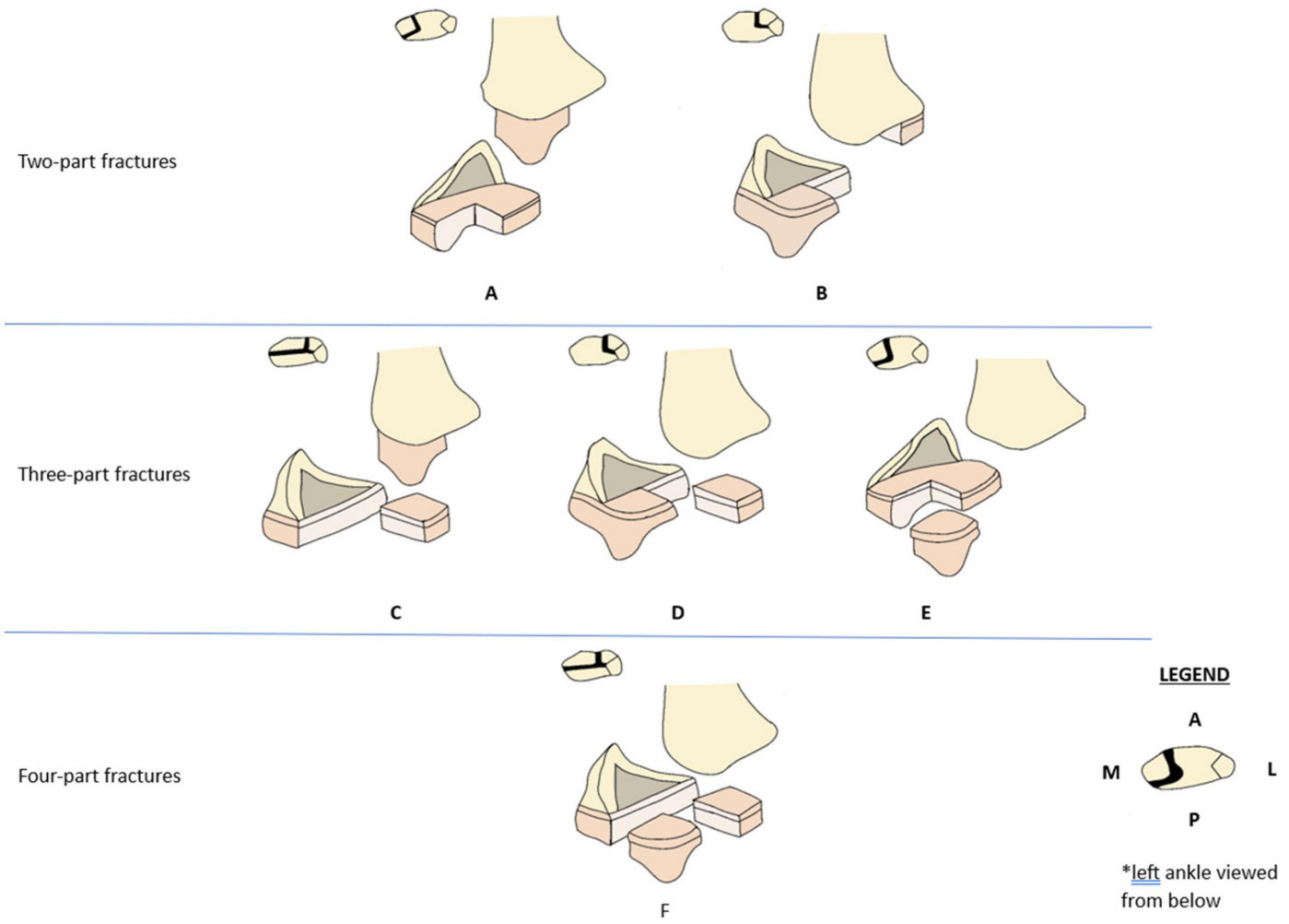
The figure illustrates the rapariz (86) classification system for triplane fractures of the distal tibia, as described by eismann et al. (63). This system is organized into three major sections. Type A and Type B represent two-part fractures, with Type A corresponding to the lateral type and Type B to the medial type. Type C, Type D, and Type E depict distinct configurations of three-part fractures. Type F denotes a four-part fracture.

**Figure 5:**
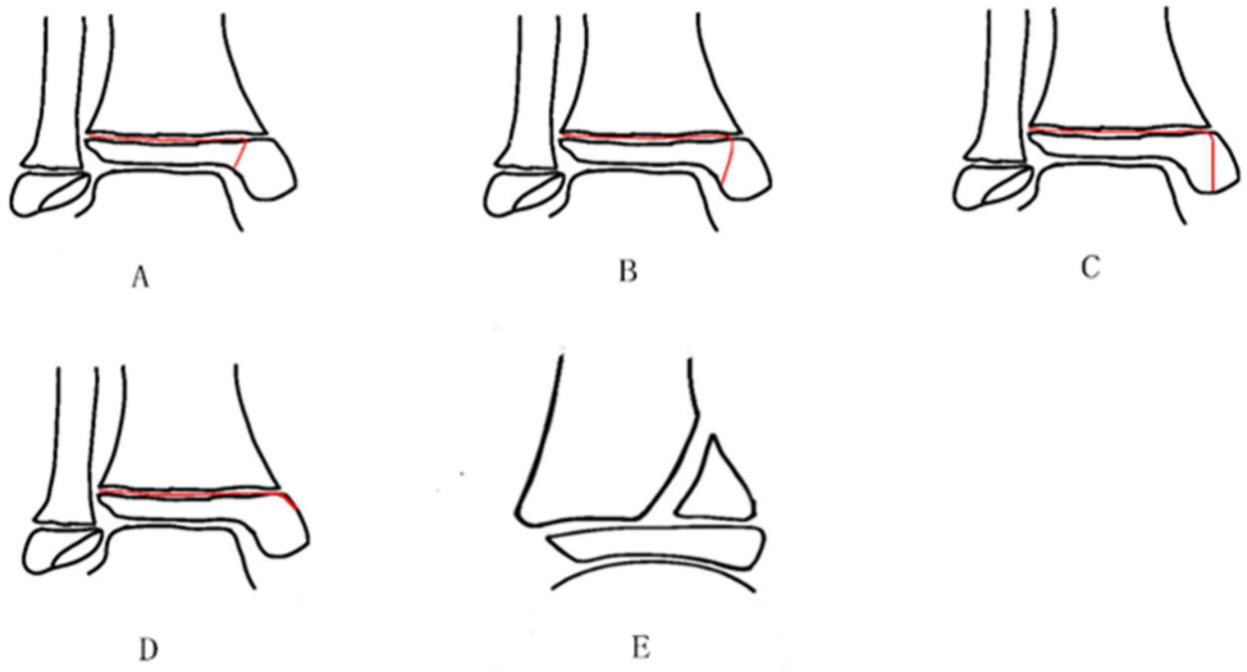
The figure illustrates the Shin-Yung classification system for atypical triplane fractures (82). This system categorizes the fractures into four primary types: Type A: Intra-articular fracture involving the weight-bearing surface; Type B: Intra-articular fracture not involving the weight-bearing surface; Type C: Extra-articular fracture; Type D: Avulsion fracture of the anteromedial epiphyseal sleeve (AMES). Type E: the AMES fracture variant.

Triplane fractures of the distal tibia were initially classified based on the number of fracture fragments, such as two-part [Cooperman (72)], three-part [Marmor (73)], and four-part [Kärrholm (74)] fractures (Figure 4). Among these, two-part fractures are the most prevalent (69). The classification based on the number of fracture fragments exhibits various morphological variants, differing in fracture morphology, number, and location (34, 75, 76). Depending on the location of the fracture line affecting the distal tibial epiphyseal plate, these fractures can be categorized into medial and lateral types. Given that the lateral portion of the distal tibial growth plate closes later than the medial portion, the lateral type is more commonly observed (77). In two-part fractures, the lateral type is predominant, with coronal fragments typically located in the posterolateral region (13). Conversely, medial-type fractures, particularly those involving the medial malleolus, are relatively rare. It was not until 1981, when Denton (78) reported the first medial malleolar triplane fracture, that this fracture type gained recognition. In 1995, Feldman (79) proposed that triplane fractures can have an extraarticular variant, distinguishing extraarticular triplane fractures from intra-articular ones, a distinction with significant implications for treatment. Building on this, Shin refined the classification of medial malleolar triplane fractures in 1997, categorizing them based on whether the fracture line involves the articular surface and weight-bearing zone: intra-articular, involving the weight-bearing surface; intra-articular, not involving the weight-bearing surface; and extra-articular (80, 81). Yung et al. (82) further revised the classification by introducing a new variant of triplane fracture—the avulsion fracture of the anteromedial epiphyseal sleeve (AMES) of the medial malleolus, characterized by a posterior metaphyseal fragment and fractures through the growth plate in the transverse plane (Figure 5). Such intra-articular fractures that do not involve load-bearing surfaces or extra-articular triplane fractures are defined as atypical triplane fractures (ATFs) (76, 82–84). Several case reports have provided further empirical evidence supporting the revision of the existing classification system (53, 85), as proposed by Yung et al.

## Treatment

A typical triplane fracture is inherently an intra-articular injury, requiring precise anatomical reduction of the epiphysis and articular surfaces to restore joint congruity. The primary objectives of treatment include minimizing complications (e.g., growth disturbances, deformities), preventing premature degenerative arthritis, and preserving long-term joint function. However, due to the anatomical complexity and relative rarity of triplane fractures, optimal treatment strategies remain a subject of debate. Current management options include non-operative treatment (Closed reduction and immobilization), open reduction and internal fixation (ORIF), closed or open reduction and percutaneous fixation, and arthroscopic-assisted surgical treatment. When evaluating a triplane fracture, two critical factors guide treatment selection: a. Articular involvement: Is the fracture type intra-articular or extra-articular? b. Displacement severity: What is the degree of intra-articular fracture displacement? These considerations ensure tailored treatment, optimizing functional recovery and reducing the risk of long-term sequelae (13).

Non-surgical treatment is indicated for triplane fractures with minimal displacement (≤2 mm) or no displacement (87). In such cases, the management approach mirrors that of single-plane fractures, immobilization combined with restricted weight-bearing can be employed. For displaced fractures that have undergone successful closed reduction, immobilization can be achieved using a cast, splint, or brace (55). Extra-articular triplane fractures are also amenable to non-surgical management, particularly those with displacement ≤5 mm, as these fractures do not necessitate strict anatomical reduction (88). Traditionally, after closed reduction, a long leg cast is widely adopted as the initial immobilization strategy for stabilizing transitional fractures of the distal tibia (89). Current evidence supports below-knee immobilization or short-leg cast is an effective alternative to long-leg casts. Neal et al. (90) conducted a comparative study of clinical outcomes in patients with transitional ankle fractures treated with either long-leg or short-leg cast immobilization. Although there were no differences in short-term outcomes and complications between the two groups of patients, the short-leg cast group demonstrated significantly shorter durations for initial plaster fixation, transition to a stabilizing brace, initiation of weight-bearing, and return to exercise compared to the long-leg cast group. These findings suggest that short-leg cast immobilization offers distinct advantages, including simplified treatment protocols, reduced risk of knee adhesions, lower psychological burden on patients, and faster rehabilitation at a lower cost. Non-surgical management is a viable option for simple triplane fractures, irrespective of the fracture count or initial displacement severity. Ryu et al. (55) conducted a retrospective study comparing clinical and imaging outcomes in 33 patients with isolated triplane fractures of the distal tibia, treated either non-operatively or surgically. With a mean follow-up of 3.5 years post-treatment, both groups demonstrated comparable and non-significant differences in American Orthopaedic Foot & Ankle Society (AOFAS) and Modified Weber-Pavlov (MWP) scores. Similarly, Ayas et al. (91) conducted a study comparing functional outcomes at 1-year post-treatment between surgically managed patients (with >2 mm displacement) and conservatively managed patients (with <2 mm displacement). While the surgically treated group demonstrated higher functional scores, the difference was not statistically significant. The “2 mm initial displacement threshold” may not be absolute in determining the choice of treatment. For fractures with >2 mm displacement, anatomical repositioning is traditionally emphasized, but conservative management remains a viable option. Importantly, residual displacement following closed reduction appears to be a more critical determinant of functional outcomes than initial displacement. Even among surgically treated patients, residual gaps after closed reduction were identified as negative prognostic indicators of functional recovery. Lurie et al. (92) evaluated the relationship between treatment modalities (conservative vs. surgical) and the degree of displacement in transitional fractures. The study followed 57 patients with transitional fractures and intra-articular gaps of 2–5 mm for at least 2 years. The findings revealed that when the intra-articular gap did not exceed 2.5 mm, conservative and surgical treatments yielded comparable efficacy. However, in conservatively managed patients with displacement >2.5 mm, mean Single Assessment Numerical Evaluation (SANE) sports scores were significantly worse. Additionally, larger intra-articular gaps correlated with poorer functional outcomes. Choudhry et al. (93) conducted a longitudinal study of 78 patients with transitional fractures, tracking postoperative functional outcomes over an average of 4.5 years. The findings demonstrated that conservative treatment yielded excellent results when residual intra-articular displacement was <2.5 mm, regardless of whether the displacement was a joint gap or a “step” deformity. Consequently, surgical intervention may be more advantageous when residual articular surface displacement exceeds 2.5 mm post-repositioning. The “2 mm” threshold for surgical indication remains controversial and requires validation through higher-quality evidence.

Indications for surgical intervention in triplane fractures include intra-articular displacement (including residual displacement) > 2 mm, extra-articular displacement >5 mm, and cases with associated combined injuries. A meta-analysis (1) revealed that males, patients with a higher number of fracture fragments, and older individuals are more frequently treated surgically. This trend may be attributed to greater male participation in high-impact sports and activities, coupled with later closure of the epiphyseal plate in males compared to females (whose bones mature earlier), increasing fracture risk. When surgery is indicated, the choice of surgical approach depends on fracture morphology and characteristics, as well as the surgeon's expertise. Surgical techniques vary, encompassing closed or open reduction, arthroscopic-assisted repositioning, and diverse internal fixation options. Closed reduction percutaneous pinning (CRPP) is the preferred minimally invasive surgical modality, aligning with contemporary orthopedic principles and offering satisfactory outcomes with reduced morbidity (94, 95). In a comparative multicenter cohort study by Zelenty et al. (24), 65 patients with transitional ankle fractures were enrolled, including 17 in the percutaneous fixation group and 48 in the ORIF group. Short-term follow-up revealed a significantly higher complication rate (19%) in the ORIF group compared to the percutaneous group. However, the limited sample size of the percutaneous fixation cohort may have introduced statistical bias, potentially compromising the reliability of the findings. Notably, most complications in the ORIF group were attributed to irritation from internal fixation hardware, increasing the likelihood of subsequent hardware removal procedures. Tang et al. (8) followed 25 patients with distal tibial triplane fractures treated with closed reduction and internal fixation (CRIF), followed for an average of 34 months. The study compared the interim clinical outcomes of two fixation methods: lag screw fixation and Kirschner wire fixation. Among all 25 children, the mean preoperative AOFAS score of 33 improved to 82 at 3 months postoperatively and 92 at final follow-up. Although premature closure of the epiphyseal plate occurred in four patients in the K-wire fixation group, neither group exhibited ankle deformity or functional limitations. Currently, limited case reports describe arthroscopic-assisted management of triplane fractures, primarily for intra-articular fractures involving the articular surface (Table 2). Arthroscopy offers direct visualization for precise anatomical reduction, particularly in knee triplane fractures with concurrent meniscal or cruciate ligament injuries, making it the preferred approach for addressing combined injuries (16). By ensuring accurate realignment and smooth joint surfaces, arthroscopy may reduce the risk of post-traumatic degenerative conditions, such as osteoarthritis (26).

**Table 2:** Review on arthroscopically assisted management of triplane fractures.

Arthroscopically Assisted Management of Triplane Fractures									
Author	Year	Gender	Age	Location of injury	Mechanism of injury	Treatment	Follow-up (months)	Complications	Outcome
Whipple (96)	1993	F	13.6	Distal tibia	Slider (baseball)	ARIF	6	None	Satisfactory
		M	16.4	Distal tibia	Sprain	ARIF	12	None	Satisfactory
Imade (97)	2004	M	14	Distal tibia	Fall	ARIF	12	None	Satisfactory
Jennings (98)	2007	F	12.4	Distal tibia	Sprain	ARIF	30	None	Satisfactory
		M	11.5	Distal Tibia + Fibula	Sprain	ARIF	36	None	Satisfactory
		M	15	Distal tibia	Sprain	ARIF	12	None	Satisfactory
		M	15.7	Distal tibia	Traffic accident	ARIF	12	None	Satisfactory
		M	15	Distal Tibia + Fibula	Fall	ARIF	48	None	Satisfactory
McGillion (54)	2007	2 M + 2 F	±13.5	Distal tibia	Traffic accident (1) Soccer ball (3)	ARIF	4∼24	None	Satisfactory
Masquijo (17)	2011	M	13	Distal femur	Motorcycle	ORIF + Arthroscopic	24	Joint adhesion and limb shortening	Satisfactory*
Strelzow (26)	2017	F	13	proximal tibia	Snowboarding	ARIF	1.5	None	Satisfactory
Whiting (16)	2021	M	13	proximal tibia	Fall	ARIF	1.5	None	Satisfactory

Only the “satisfactory*” patient had complications of joint adhesion and limb shortening due to severe injury. However, satisfactory results can still be achieved after arthroscopic release. Satisfactory outcome defined when the patient can return to normal activities and no functional limitations. ARIF, arthroscopically assisted reduction and internal fixation; ORIF: open reduction and internal fixation.

In summary, when a triplane fracture occurs, closed reduction can be initially attempted as a liable treatment option to correct the initial displacement. However, conversion to incisional reduction surgery should be considered when more complex factors such as poor closed reduction, large residual displaced deformity, and fracture instability are present. Residual displacement may cause chronic joint pain and premature osteoarthritis due to articular surface incongruity. Soft tissue interposition, such as periosteal entrapment (especially at the anterolateral corner of the distal tibia) or perichondral ring, often complicates closed reduction of triplane fractures (99). Additionally, periosteal entrapment of the anterolateral corner of the distal tibia, combined with fibula fractures and severe displacement are independent risk factors for requiring ORIF (88). In cases of complex triplane fractures (e.g., severe displacement, multiple fracture fragments, or soft tissue interposition), thorough preoperative assessment is essential. CT imaging should be used to accurately assess the number and configuration of fracture fragments and quantify the degree of displacement and articular surface involvement. This information enables the formulation of a personalized treatment plan tailored to the fracture's specific characteristics. According to the different characteristics of the fracture, the surgical incision and screw placement position should be reasonably selected to achieve anatomical reduction, stable fixation and preservation of joint function. This tailored approach, at the same time, provides a reference framework for orthopedic surgeons managing complex cases. For example, for distal tibia triplane fracture with periosteal interposition, it is recommended to choose an anterolateral surgical incision to simplify the surgical procedure and realize anatomical reduction at the same time (71). Different types of internal fixation may not have a substantial impact on treatment outcomes. The use of either Kirschner wires or cannulated screws for the fixation of distal tibial transitional fractures has demonstrated comparable efficacy. In a study conducted by Mishra et al. (100), 49 patients were divided into two groups: 18 patients in the Kirschner wire fixation group and 31 patients in the screw fixation group. The aim was to compare the clinical efficacy and radiographic outcomes of these two different internal implants following open reduction. According to the modified Weber scoring system, at the final follow-up, both groups exhibited excellent clinical and radiographic results, with complete fracture healing and no residual displacement observed. Additionally, there was no significant difference in the time required for patients to resume physical activities between the two groups. Although the operative time was significantly shorter in the Kirschner wire fixation group, it necessitated a longer postoperative period of immobilization with cast.

When it comes to triplane fractures occurring at other anatomical sites, there exists a dearth of diagnostic criteria and an established optimal treatment approach, primarily due to the rarity of such cases. However, given the similarity in fracture patterns, it is feasible to draw inspiration from the treatment protocols designed for triplane fractures of the distal tibia. It is crucial to acknowledge, though, that closure pattern of the epiphyseal plate of triplane fractures can vary across different sites, and these fractures may exhibit distinct characteristics and variations. This underscores the importance of adhering to the principle of individualized treatment when determining the most suitable surgical approach in clinical settings. Moreover, a comprehensive understanding of the closure pattern of the epiphyseal plate at each specific site can significantly aid in surgical decision-making. For instance, if closure of the epiphyseal plate is characterized by a homogeneous pattern, functionally, when the epiphyseal plate is partially closed, it can be inferred that the entire epiphyseal growth plate is effectively closed, thereby minimizing the risk of growth disturbances. This insight can guide intraoperative decisions, such as the choice of an anterior cruciate ligament reconstruction technique utilizing a trans-epiphyseal tunnel. While premature closure of the epiphyseal plate and partial growth arrest are potential complications associated with these rare fractures, they are generally not severe enough to cause significant growth-related issues. Triplane fractures typically manifest during the final stages of skeletal maturation and growth arrest in adolescents, a period when growth has essentially ceased. Consequently, the prognosis for these fractures is usually favorable, with no discernible correlation between the complication rate and the type of treatment employed (1).

## Conclusion

Given the unique characteristics of triplane fractures, including their age-specific occurrence (typically affecting adolescents) and complex anatomical involvement (often spanning the distal tibial physis and metaphysis), CT imaging is strongly recommended for patients with suspected triplane fractures. CT enables comprehensive evaluation of fracture comminution, displacement, and articular surface involvement, thereby minimizing the risk of missed or incorrect diagnoses. Treatment strategies should be individualized, accounting for fracture patterns, status of physeal closure, and associated injuries. However, current research on triplane fractures is predominantly focused on distal tibial injuries and consists largely of retrospective studies with limitations such as small sample sizes, short follow-up durations, and inconsistent imaging or outcome assessments. Additionally, there is a scarcity of data on fractures in non-tibial locations or long-term functional outcomes. To address these gaps, high-quality prospective studies are warranted to establish evidence-based, standardized treatment algorithms. Such research would better inform clinical decision-making and improve outcomes for patients with triplane fractures across diverse anatomical regions.
